# Durable Clinical Response to ALK Tyrosine Kinase Inhibitors in Epithelioid Inflammatory Myofibroblastic Sarcoma Harboring *PRRC2B-ALK* Rearrangement: A Case Report

**DOI:** 10.3389/fonc.2022.761558

**Published:** 2022-02-14

**Authors:** Zhan Wang, Yan Geng, Ling-Yan Yuan, Miao-Miao Wang, Chen-Yang Ye, Li Sun, Wei-Ping Dai, Yuan-Sheng Zang

**Affiliations:** ^1^Department of Medical Oncology, Second Affiliated Hospital of Naval Medical University, Shanghai, China; ^2^Department of Nursing, Shanghai Jing’an District Zhabei Central Hospital, Shanghai, China

**Keywords:** epithelioid inflammatory myofibroblastic sarcoma, IMT, *PRRC2B-ALK*, *ALK* R1192P, *ALK* L1196M, crizotinib resistance, alectinib, ceritinib

## Abstract

Inflammatory myofibroblastic tumor (IMT) is a rare mesenchymal neoplasm and patients with IMT tend to have a favorable outcome after complete surgical resection. However, some tumors of IMT cases have recurred and grown rapidly after successful surgery. Epithelioid inflammatory myofibroblastic sarcoma (EIMS) is a highly aggressive intra-abdominal IMT variant with epithelioid-to-round cell morphology. Currently, no standard therapy exists for recurrent or invasive IMTs and EIMS, but anaplastic lymphoma kinase (ALK) tyrosine kinase inhibitors (TKIs) are recommended for those harboring *ALK* gene rearrangements. We herein report the first case of *PRRC2B-ALK* fusion associated IMTs with clinical and pathological manifestation matched the diagnosis criteria of EIMS and the durable clinical response of the sequential use of ALK TKIs (crizotinib, alectinib, ceritinib, and lorlatinib). A female patient with EIMS of the greater omentum was suffering from a rapid recurrence after cytoreductive surgery was done. Crizotinib was administered when *PRRC2B-ALK* fusion was detected, and partial response was achieved. The progression-free survival (PFS) of crizotinib was 5 months. Alectinib was administered based on the results of second next-generation sequencing (NGS) analysis, which identified the secondary mutation *ALK* R1192P. The best overall response of alectinib treatment was a partial response (PR) and the PFS was 5.5 months. Ceritinib was prescribed as third-line therapy after alectinib resistance with *ALK* L1196M mutation. PR was achieved and the PFS of ceritinib was 6 months. The patient was taking lorlatinib after ceritinib resistance and achieved a stable disease at 2 months with the PFS more than 5 months. The overall survival was more than two years as of the time of manuscript preparation. We describe an EIMS of greater omentum caused by *PRRC2B-ALK* fusion gene and showed durable clinical response to the sequential use of ALK TKIs.

## Introduction

Inflammatory myofibroblastic tumor (IMT) is extremely rare and characterized by the proliferation of myofibroblastic spindle cells with varying extent of inflammatory cell infiltrates (1). Most patients with IMT tend to have a favorable outcome after complete surgical resection. Similar to *anaplastic lymphoma kinase (ALK)* fusion in non-small cell lung cancer (NSCLC), *ALK* rearrangements in IMT results in protein overexpression and activation of its kinase domain that drives cancer development and progression (2–4). Rearrangements involving the *ALK* gene located at chromosome 2p23 have been reported in 40%–60% of IMT cases (5–7). Partner genes of *ALK* including *NPM, TPM3, TPM4, CLTC, RANBP2, CARS, ATIC, SEC31L1, EML4, TFG, LMNA, FN1, PPFIBP2, DCTN1* and *RRBP1* have been reported (8). The relationship between the particular ALK partner and the morphology or prognosis of IMT remains unknown.

In some IMT cases, the tumors have recurred and grown rapidly after successful surgery. Epithelioid inflammatory myofibroblastic sarcoma (EIMS) is a highly aggressive intra-abdominal IMT variant with epithelioid-to-round cell morphology and nuclear membrane or perinuclear ALK staining (9). *RANBP2-ALK* fusion is one of the key drivers of EIMS which experiences early disease recurrence and poor prognosis (9). Recently, *EML4-ALK* fusion is reported as an alternative fusion gene in EIMS (8). *PRRC2B-ALK* was previously reported in only one patient with pediatric subependymal giant cell astrocytoma (SEGA) (10) but never in IMT or EMIS patients.

Herein, we described the case of a patient with *PRRC2B-ALK* associated IMT of the greater omentum, whose clinical and pathological manifestation matched the diagnosis criteria of EIMS. The patient benefited from the sequential use of ALK TKIs (crizotinib, alectinib, ceritinib, and lorlatinib).

## Case Description

**Figure 1:**
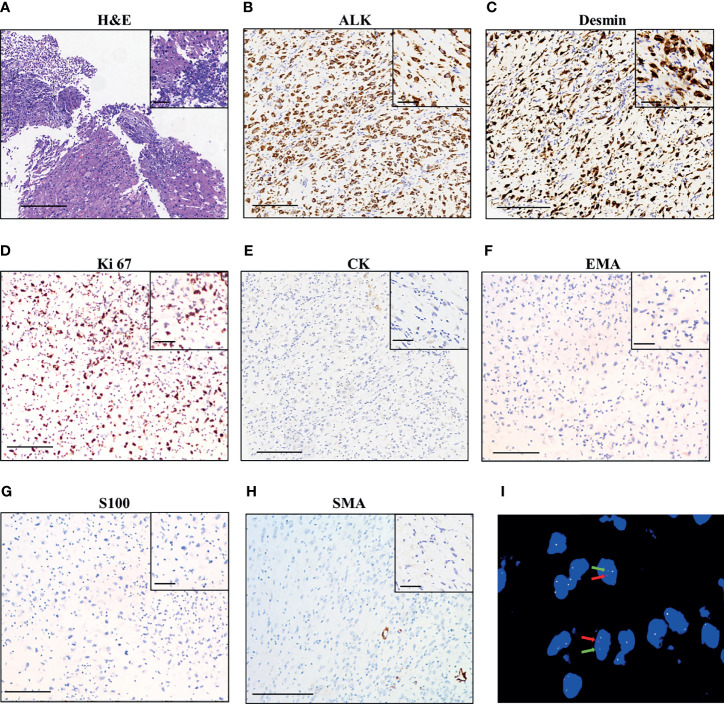
Pathological findings of the patient. **(A)** Hematoxylin–eosin staining of surgical tumor sample. IHC staining of ALK **(B)**, desmin **(C)**, Ki 67 **(D)**, CK (Cytokeratin, **(E)**, EMA **(F)**, S100 **(G)**, and SMA **(H)**. The magnification in **(A–H)** is 100×, scale bar is 200 μm; the magnification in the inset images is 400×, scale bar is 50 μm. **(I)** Fluorescence *in situ* hybridization (FISH) using a break-apart ALK locus probe. ALK gene rearrangement is indicated by the split signals (indicated by red and green arrows, magnification 1000×).

A 42-year-old Chinese woman was admitted to a local hospital with a history of abdominal distention and intermittent abdominal pain. The contrast-enhanced computed tomography (CT) scan of the abdomen identified an enlarged pelvic mass with peritoneal metastasis, which was suspected as ovarian carcinoma. Cytoreductive surgery was then performed in November 2019, which removed the tumor in the greater omentum with a volume of 19 × 19 × 10 cm. No visible tumors were seen in both ovaries or fallopian tubes. The histopathological examination of the surgical specimen showed the lesion consisted of both epithelioid and spindle cells with nuclear atypia and inflammatory cells infiltration, mainly neutrophils (**Figure 1A**). Immuno-histochemistry (IHC) was positive for ALK p80 (**Figure 1B**), desmin (**Figure 1C**) and Ki 67 (30% +, **Figure 1D**), and negative for Cytokeratin (CK, **Figure 1E**), EMA (**Figure 1F**), Actin, CD117, S-100 (**Figure 1G**), SOX-10, SMA (**Figure 1H**), CD34, and STAT6. These findings are consistent with IMT. The patient did not receive adjuvant therapy after surgery.

**Figure 2:**
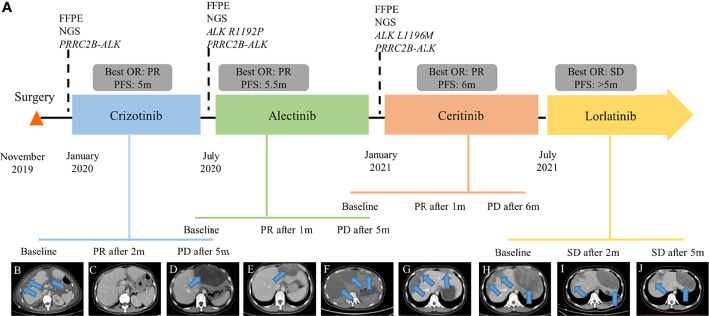
Clinical summary of the patient. **(A)** Therapeutic timeline of the patient. Abdominal CT radiograph at baseline before crizotinib therapy **(B)**, 2 months of crizotinib therapy **(C)**, 5 months of crizotinib therapy **(D)**, 1 month of alectinib treatment **(E)**, 5 months of alectinib treatment **(F)**, 1 month of ceritinib treatment **(G)**, 6 months of ceritinib treatment **(H)**, 2 months of lorlatinib treatment **(I)**, and 5 months of lorlatinib treatment **(J)**. FFPE, formalin-fixed, paraffin-embedded; NGS, next-generation sequencing; OR, overall response; PFS, progression-free survival; PR, partial response; PD, progression disease; SD, stable disease. The arrows indicate the tumor lesions and ascites.

**Figure 3:**
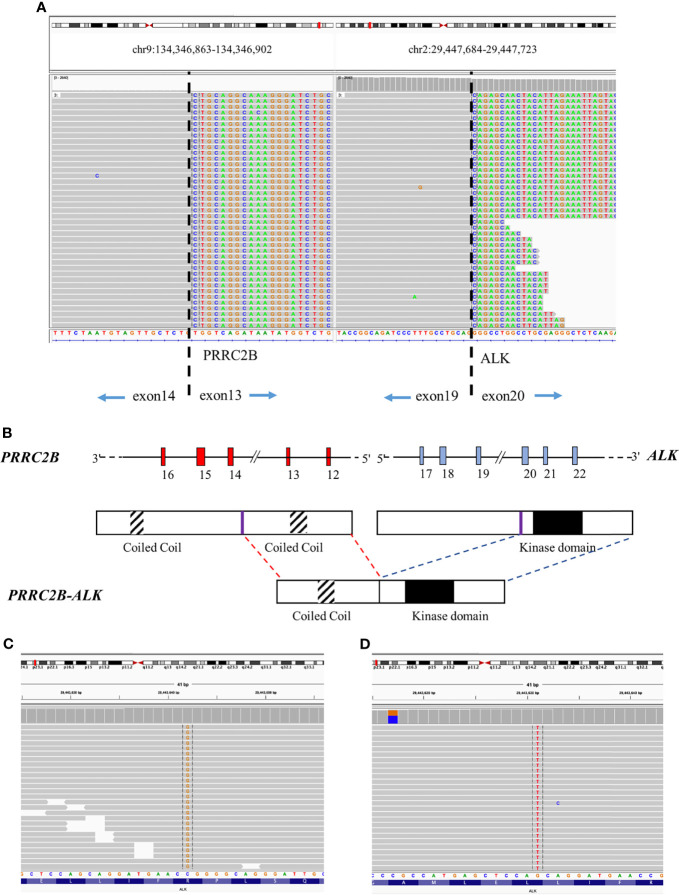
Sequencing reads of *PRRC2B* and *ALK* visualized by the Integrative Genomics Viewer (IGV). **(A)** Next-generation sequencing identified *PRRC2B*-*ALK* fusion before crizotinib treatment. **(B)** A schematic map showing the structure of the *PRRC2B*-*ALK* fusion locus. The slash marks on the introns indicate the break points. Next-generation sequencing identified *ALK* R1192P after crizotinib resistance **(C)** and *ALK* L1196M after alectinib resistance **(D)**.

The patient was referred to our hospital due to abdominal distension, pain, and bloating 40 days after surgery. Fluorescence *in situ* hybridization (FISH) of the surgical specimen showed positive results for *ALK* fusion (**Figure 1I**). Two independent in-house pathology consultants favored the diagnosis of EIMS, a recently defined variant of IMT. The patient’s therapeutic course and radiological examinations are summarized in **Figure 2A**. CT showed the presence of ascites and multiple lesions in the abdominal cavity (**Figure 2B**), indicating tumor recurrence. To explore cancer-related genetic alterations, next-generation sequencing (NGS) was performed on the archived surgical tumor samples using a targeted panel consisting of 520 cancer-related genes (Burning Rock Biotech, Guangzhou, China). The result revealed a rare fusion involving the exons 1–13 of *PRRC2B* located at chromosome 9q34 and exons 20–29 of *ALK* located at chromosome 2p23 (P13; A20) (**Figure 3A**). The kinase domain of ALK and the coiled-coil domain of its fusion partner, PRRC2B, were retained (**Figure 3B**). The patient was subsequently treated with first-generation ALK TKI crizotinib (250 mg, bid) as the first-line therapy. Two months later, her symptoms significantly improved. The CT scan showed a remarkable reduction of the ascites (**Figure 2C**). The efficacy of the first-line crizotinib treatment was assessed as partial response (PR) according to the Response Evaluation Criteria in Solid Tumors (RECIST) (11). The progression-free survival (PFS) was 5 months, then she developed abdominal pain and abnormal liver function. Follow-up CT imaging showed a low-density mass in the abdomen (**Figure 2D**), suggesting disease progression. Crizotinib was discontinued.

To identify the mechanism underlying resistance to crizotinib, intra-abdominal re-biopsy was performed and pathologically confirmed EIMS. NGS detected the emergence of *ALK* R1192P (**Figure 3C**) and retention of *PRRC2B-ALK* fusion. Subsequently, she was switched to a next-generation ALK TKI alectinib at a dose of 600 mg twice daily. After 1 month treatment, her abdominal pain was significantly alleviated with the peritoneal lesion regressed rapidly by 35.7% per RECIST (**Figure 2E**). The best response of alectinib treatment was PR and the PFS was 5.5 months (**Figure 2F**). Alectinib treatment was discontinued due to disease progression.

New intra-abdominal biopsy was performed and NGS detected the emergence of *ALK* L1196M (**Figure 3D**) and retention of *PRRC2B-ALK* fusion. Ceritinib (450 mg/d) was administered, resulting in remarkable shrinkage of the primary lesion, and achieving a PR within a month (**Figure 2G**). Follow-up CT showed disease progression after 6 months’ treatment (**Figure 2H**). Ceritinib was then switched to lorlatinib (100 mg/d) on July 2021 and stable disease (SD) was achieved after 2 months (**Figure 2I**) and confirmed after 5 months of lorlatinib treatment (**Figure 2J**). The patient was in stable condition as we prepared the manuscript. There were no significant adverse events throughout the course of treatment.

## Discussion

In this case we have reported a case of *PRRC2B-ALK* fusion associated IMTs with clinical and pathological manifestation matched the diagnosis criteria of EIMS and the benefit of the sequential use of ALK TKIs (crizotinib, alectinib, ceritinib, and lorlatinib). *ALK* R1192P and *ALK* L1196M mutations were identified by NGS, suggesting a potential association between the mutations and resistance to ALK TKIs. The OS is more than two years as of the time of manuscript preparation.

In 2011, Marino-Enriquez et al. first named and described in detail the clinicopathological, immunohistochemical and genetic characteristics of EIMS to highlight both the distinct morphology and malignant behavior of this aggressive form of IMT (9). EIMS is usually intra-abdominal and highly aggressive with epithelioid-to round cell morphology, and prominent inflammatory infiltrate. For IHC, tumors were all positive for ALK, and mostly positive for desmin. Focal reactivity for SMA was detected in half of the cases, including the desmin-negative case (9). In our case, desmin was positive but SMA was negative. Genetically, *RANBP2-ALK* fusion was the most reported driver mutation of EIMS (9, 12–15). *EML4-ALK* associated IMTs have recently been categorized as EIMS (8).

*PRRC2B-ALK* was previously reported in only one patient with pediatric subependymal giant cell astrocytoma (SEGA), and may play a key oncogenic role in the SEGA tumor (10). As we prepared the manuscript, Gupta et al. reported the first *PRRC2B-ALK* fusion in EIMS of the omentum, which was the same diagnosis as our patient (16). Despite an initially promising therapeutic response, the patient died partially due to significant diagnostic challenges and a lack of timely targeted therapy (16). In the present case, as observed in other recurrent ALK fusions (16, 17), the kinase domain of ALK and the coiled-coil domain of its fusion partner, PRRC2B, were retained. Oligomerization *via* the coiled-coil domain leads to constitutive kinase activation. Considering the patient responded to crizotinib therapy within 2 months, *PRRC2B-ALK* fusion might be the main oncogenic driver of the EIMS in our patient.

The optimal therapy for EIMS has not been well established. Radical surgery is the standard treatment for patients with localized IMT, but no standard therapeutic modality is available for recurrent or invasive IMT (7, 18). The National Comprehensive Cancer Network guidelines recommend the use of ALK TKIs in *ALK*-positive IMTs. Dramatic and durable responses to crizotinib have been observed in ALK-positive IMT patients (19–21). Disease relapse during ALK inhibitor treatment occurs in ALK-rearranged IMT (20, 22). In our case, the patient’s previous clinical symptoms were significantly relieved after each administration of ALK TKIs, however, for a short time only.

Long-term effectiveness of ALK inhibitors, including crizotinib, ceritinib, and alectinib, can be limited by ALK resistance mutations that emerge during treatment (23). The first *ALK* resistance mutation reported was the L1196M gatekeeper mutation (24), which generates resistance to crizotinib but then becomes sensitive to many of the next-generation inhibitors, including ceritinib, alectinib, and brigatinib. In our case, *ALK* L1196M was acquired after alectinib resistance and conferred sensitivity to ceritinib.

The *ALK* R1192P mutation was first reported in a patient with incompletely penetrant neuroblastic tumor (25), but never in IMT or EIMS patients. *ALK* R1192P induces the gain-of-function of ALK protein, leading to increased downstream signaling (26, 27). *ALK* R1192P *in cis* configuration with G1202R was detected in a heavily treated patient with lung adenocarcinoma, who had a dramatic response to lorlatinib, a third-generation ALK inhibitor (28). Since G1202R is a well-understood resistance mechanism for first- and second-generation ALK inhibitors (29–31), it is difficult to identify the specific role of R1192P in mediating ALK TKIs resistance and its sensitivity to next-generation ALK inhibitors. In our case, the administration of alectinib successfully reversed the crizotinib resistance based on the rapid improvement in the patient’s clinical symptoms coupled with radiological response, which provided clinical evidence that *ALK* R1192P mutation was a driver of crizotinib resistance and conferred sensitivity to alectinib.

The molecular testing of *ALK* fusion is essential for all patients diagnosed with EIMS to identify those who are likely to benefit from ALK TKIs treatment, particularly for patients who relapse on a second-generation ALK TKI (32). On disease progression, NGS of the repeat biopsy sample also helps uncover the mechanisms of acquired resistance. Our study found *ALK* R1192P as the potential mechanism of crizotinib resistance, and the efficacy of alectinib in overcoming *ALK* R1192P-mediated crizotinib resistance. It highlighted the importance of NGS in identifying actionable mutations and resistance mechanisms that could guide the use of molecular targeted drugs for the effective management of EIMS with *ALK* gene arrangement.

## Conclusion

We report the case of *PRRC2B-ALK* fusion associated EIMS and describe the durable clinical response to sequential use of ALK TKIs (crizotinib, alectinib, ceritinib, and lorlatinib). Moreover, we reveal that *ALK* R1192P and L1196M mutations are acquired after ALK TKIs resistance in EIMS patients. Our clinical evidence suggests that *ALK* R1192P mutation is a driver of crizotinib resistance. It highlights the importance of NGS in identifying actionable mutations and resistance mechanisms that could guide the use of molecular targeted therapies for the effective management of EIMS with ALK gene arrangement. The therapeutic strategies used in this case could serve as a treatment reference for EIMS patients with ALK rearrangements and acquired inhibitor resistance through the course of treatment.

## Data Availability Statement

The original contributions presented in the study are included in the article/supplementary material. Further inquiries can be directed to the corresponding author.

## Ethics Statement

Written informed consent was obtained from the individual for the publication of any potentially identifiable images or data included in this article.

## Author Contributions

ZW and YZ conceived and designed the study. ZW, YG, LY, MW, and CY collected the clinical data and reviewed the literature. ZW, YG, LS, and WD analyzed and interpreted the data. ZW drafted the manuscript. All authors contributed to the article and approved the submitted version.

## Funding

This work was supported by grants from Medical Innovation Research Project of Shanghai Science and Technology Commission (No. 20Y11914400, 2020).

## Conflict of Interest

The authors declare that the research was conducted in the absence of any commercial or financial relationships that could be construed as a potential conflict of interest.

## Publisher’s Note

All claims expressed in this article are solely those of the authors and do not necessarily represent those of their affiliated organizations, or those of the publisher, the editors and the reviewers. Any product that may be evaluated in this article, or claim that may be made by its manufacturer, is not guaranteed or endorsed by the publisher.
